# Triglyceride associated polymorphisms of the *APOA5 *gene have very different allele frequencies in Pune, India compared to Europeans

**DOI:** 10.1186/1471-2350-7-76

**Published:** 2006-10-10

**Authors:** Giriraj R Chandak, Kirsten J Ward, Chittaranjan S Yajnik, Anand N Pandit, Ashish Bavdekar, Charu V Joglekar, Caroline HD Fall, P Mohankrishna, Terence J Wilkin, Bradley S Metcalf, Michael N Weedon, Timothy M Frayling, Andrew T Hattersley

**Affiliations:** 1 Genome Research Group, Centre for Cellular and Molecular Biology, Hyderabad, India; 2 Institute of Biomedical and Clinical Science, Peninsula Medical School, Exeter, UK; 3 Diabetes Unit, King Edward Memorial Hospital and Research Centre, Pune, India; 4 Department of Pediatrics, King Edward Memorial Hospital and Research Centre, Pune, India; 5 Medical Research Council, Epidemiology Resource Centre, Southampton, UK; 6 Departments of Endocrinology and Metabolism, Peninsula Medical School, Plymouth, UK

## Abstract

**Background:**

The *APOA5 *gene variants, -1131T>C and S19W, are associated with altered triglyceride concentrations in studies of subjects of Caucasian and East Asian descent. There are few studies of these variants in South Asians. We investigated whether the two *APOA5 *variants also show similar association with various lipid parameters in Indian population as in the UK white subjects.

**Methods:**

We genotyped 557 Indian adults from Pune, India, and 237 UK white adults for -1131T>C and S19W variants in the *APOA5 *gene, compared their allelic and genotype frequency and determined their association with fasting serum triglycerides, total cholesterol, HDL and LDL cholesterol levels using univariate general linear analysis. *APOC3 SstI *polymorphism was also analyzed in 175 Pune Indian subjects for analysis of linkage disequilibrium with the *APOA5 *variants.

**Results:**

The *APOA5 *-1131C allele was more prevalent in Indians from Pune (Pune Indians) compared to UK white subjects (allele frequency 20% vs. 4%, p = 0.00001), whereas the 19W allele was less prevalent (3% vs. 6% p = 0.0015). Patterns of linkage disequilibrium between the two variants were similar between the two populations and confirmed that they occur on two different haplotypes. In Pune Indians, the presence of -1131C allele and the 19W allele was associated with a 19% and 15% increase respectively in triglyceride concentrations although only -1131C was significant (p = 0.0003). This effect size was similar to that seen in the UK white subjects. Analysis of the *APOC3 SstI *polymorphism in 175 Pune Indian subjects showed that this variant is not in appreciable linkage disequilibrium with the *APOA5 *-1131T>C variant (r^2 ^= 0.07).

**Conclusion:**

This is the first study to look at the role of *APOA5 *in Asian Indian subjects that reside in India. The -1131C allele is more prevalent and the 19W allele is less prevalent in Pune Indians compared to UK Caucasians. We confirm that the *APOA5 *variants are associated with triglyceride levels independent of ethnicity and that this association is similar in magnitude in Asian Indians and Caucasians. The -1131C allele is present in 36% of the Pune Indian population making it a powerful marker for looking at the role of elevated triglycerides in important conditions such as pancreatitis, diabetes and coronary heart disease.

## Background

The *APOA5 *gene was first identified as a conserved DNA sequence 27 Kb proximal to the Apolipoprotein cluster of *APOA1, APOA4 *and *APOC3 *on chromosome 11q23 [1]. It is an important regulator of triglycerides, as established by both genetic manipulation in animals [1] and a large number of genetic studies in man [1-8]. To date, most studies of the *APOA5 *gene have been conducted in cohorts of Caucasian subjects and have shown that two haplotypes, defined by the SNPs -1131T>C and S19W, are independently associated with triglyceride concentrations. The association between -1131T>C and triglyceride concentrations is likely to be explained by linkage disequilibrium (LD) with SNPs in the neighbouring *APOC3 *gene whilst there is no LD between S19W and *APOC3 *variants [9]. Supporting this, the -1131C allele is associated with lower APOA5 concentrations and higher triglyceride concentrations whereas the 19W allele is associated with higher APOA5 and higher triglyceride concentrations, relative to subjects homozygous for the less common allele [10,11]. The majority of published reports have observed the minor allele frequency for both these variants to be approximately 6% in Caucasian populations [1-8]. However, the frequencies vary considerably across different ethnic groups. The prevalence of -1131C allele varies between 6.4% in American Caucasian males [5] and 34% in the Japanese population [12], whilst the 19W allele frequency varies between 0.1% in a Chinese population [13] and 15.8% in Hispanic males [5]. The reported size of effect of the less common alleles on triglyceride concentration varies in different cohorts from different races [2,5,12-15]. However, most studies show a 15–30% increase in triglyceride concentrations with the inheritance of the minor allele at these polymorphisms and there is no clear evidence that ethnic origin determines the size of the increase.

There is limited data available on the *APOA5 *gene variants in the Asian Indian population. The only study to date is on Asian Indians residing in Singapore and shows a minor allele frequency of 23% at -1131T>C and <3% for the S19W polymorphisms [13]. No study has been conducted on the role of *APOA5 *in Asian Indians residing in India. It is important to understand the genetic contribution to triglyceride metabolism in Asian Indians since they have higher triglyceride concentrations compared to Caucasians relative to their BMI and these differences can occur as early as 10 years old [16].

In this study, we have compared the allele frequency of the -1131T>C and S19W variants in Pune Indians and UK white subjects and examined the size of their effect on fasting triglycerides and other lipid parameters in these two populations.

## Methods

### Subjects

The Asian Indian adult cohort comprised of 557 parents from the Pune children study, which is a consecutive birth cohort from KEM Hospital, Pune [17]. This cohort consisted of subjects of Indo European descent from Maharashtra in Western India. The Institutional Ethics Committee approved the study following the Indian Council of Medical Research guidelines for research on human subjects. All subjects gave written informed consent to participate in the study. The UK white adult cohort included 237 parents from the Plymouth EarlyBird study, which is a non-intervention prospective study of school age children and their parents [18]. This cohort complies with the Declaration of Helsinki, and Local Research Ethics Committee approval was obtained early in 1999. Subjects with diabetes on fasting and 2-hour OGTT criteria were excluded. Clinical details of subjects are given in table 1.

**Table 1:** Clinical characteristics of Pune Indian individuals and UK white subjects.

	Pune Indians	UK Whites
N	557	237
% male	45	52
Age (Yrs)	36 (32–40)	34 (31–38)
BMI (kg/m^2^)	22.3 (19.7–24.9)	26 (23.2–28.6)
Total Cholesterol (mg/dl)	155 (133 – 184)	187 (168 – 215)
HDL-Cholesterol (mg/dl)	40 (32 – 49)	53 (46 – 64)
LDL-Cholesterol (mg/dl)	94 (77 – 118)	113 (93 – 139)
Triglycerides (mg/dl)	85 (59 – 119)	84 (62 – 117)
Fasting glucose (mg/dl)	88 (80–96)	85 (79–88)
Fasting insulin (mg/dl)	34 (22–50)	53 (42–75)

Pune Indian individuals (adults from the Pune Children Study) and UK white subjects (adults from the Plymouth EarlyBird study).

Data presented as median (interquartile range); BMI; Body Mass Index, HDL; High Density Lipoprotein, LDL; Low Density Lipoprotein.

### Genotyping APOA5 and APOC3 variants

The UK white subjects were genotyped for -1131T>C polymorphism using the RFLP assay with 2 U of *MseI *(New England Biolabs, UK), as previously described [7] while, genotyping of Pune Indian individuals was performed using a tetra-primer based PCR method, previously described [19]. Both the cohorts were genotyped for S19W using an RFLP protocol with 7 U of *EagI*, as previously described [7]. The results of tetra primer assay for -1131T>C were validated by RFLP analysis in ~10% (50/524) of randomly chosen Pune Indian samples. In an attempt to investigate the extent of LD between the *APOA5 *-1131T>C and the *APOC3 SstI *variants, we genotyped the *APOC3 SstI *SNP in 175 Pune Indians by RFLP assay using 10 U of *SstI *[7] and validated the results in ~15% (30/175) samples by sequencing.

### Quality control measures

Two independent individuals scored the agarose gels to determine the genotype and any discrepancies were resolved by sequencing. Rare homozygotes were confirmed by re-genotyping and 10% of randomly selected samples were re-genotyped with a genotyping accuracy of >99% for all the variants. Overall, genotypes were obtained for all the UK white adult samples and 94% (n = 524) of Pune Indian adults.

### Statistics

Allele and genotype frequencies were calculated for both SNPs using chi-squared analyses. Hardy-Weinberg Equilibrium (HWE) was tested by comparing the observed with the expected genotype frequency using Chi-square analysis. The LDMAX function of GOLD software was used to calculate the linkage disequilibrium between the two SNPs in *APOA5 *gene and between the *APOA5 *-1131T>C SNP and *APOC3 SstI *variant. Association analysis between the genotype and various lipid parameters was performed using univariate general linear model analysis in SPSS (SPSS 11.0). Continuous parameters were log transformed where necessary before conducting any analysis. Genotypes were tested for association with potential covariates of triglyceride concentrations and if significantly associated, genotype-triglyceride comparisons were adjusted. Since the homozygotes for the less common allele were rare (<0.05) for both the *APOA5 *polymorphisms, the heterozygotes and the less common allele homozygotes were pooled for further analysis: ((TC+CC) and (SW+WW)). The independent T-Test was used to determine the standardized effect of these variants on triglyceride concentrations with the presence or absence of the less common allele. Regression analysis was performed to determine the independent effect of different variables on triglyceride concentrations. Although statistical tests used logged variables where necessary, we present untransformed means for consistency with previous studies.

## Results and discussion

We present the first study investigating the common *APOA5 *variants and their association with lipid concentrations in Asian Indian adults, residing in Pune, India, as compared to UK white adults.

### Ethnic differences in the allele frequency of the -1131T>C and the S19W polymorphisms

A total of 761 individuals comprising 524 Pune Indian adults and 237 UK white adults were successfully genotyped for the -1131T>C and S19W polymorphisms in the *APOA5 *gene. The less common allele at -1131T>C variant was significantly more prevalent than the S19W polymorphism in the Pune Indians (20% vs. 3% respectively), whereas the allele frequency for both SNPs was comparable in UK white subjects (4% and 6% respectively: table 2). We observed a marked difference in the minor allele frequency at both SNPs between the Pune Indians and UK white subjects (table 2). The -1131C allele is more prevalent in the Pune Indian subjects compared to the UK white subjects (allele frequency 20% vs. 4%, p = 0.00001), while the 19W allele is less prevalent in the Pune Indians (allele frequency 3% vs. 6% p = 0.0015). Approximately, 1/3^rd ^(36%) of Indian subjects carried at least one copy of the less common allele at -1131T>C compared to only 8% of UK white individuals but only 5% of Pune Indian individuals carry the 19W allele compared to 11% UK white subjects.

**Table 2:** Prevalence of *APOA5 *gene variants -1131T>C and S19W in Asian Indians and UK white subjects.

SNP	-1131T>C					S19W				
	
	TT	TC	CC	Carrier* Frequency (95% CIs)	Less common allele Frequency (95% CIs)	SS	SW	WW	Carrier* Frequency (95% CIs)	Less common allele Frequency (95% CIs)
Pune Indians (n = 524)	336	170	18	0.36 (0.32–0.40)	0.20 (0.17–0.22)	497	27	0	0.05 (0.03–0.07)	0.03 (0.02–0.04)
UK whites (n = 237)	218	19	0	0.08 (0.05–0.11)	0.04 (0.02–0.06)	210	26	1	0.11 (0.07–0.15)	0.06 (0.04–0.08)
P value					<0.00001					0.0015

Asian Indians (adults from the Pune Children Study) and UK white subjects (adults from the Plymouth EarlyBird study).

P values are calculated for differences in genotype frequencies between Pune Indians and UK white subjects. *Carrier frequency indicates individuals carrying at least one copy of the less common allele at either polymorphism. Neither population showed any deviation from HWE for either polymorphism (p > 0.05).

The allele frequencies in Pune Indians are consistent with the only previous study of individuals of Indian descent from Singapore (23% for 1131C allele and <3% for 19W allele) [13]. This follows a similar pattern in other Asian ethnic groups such as Japanese, Malaysian and Chinese which also have a higher prevalence of the -1131C allele [13] in contrast to Caucasian populations where the two variants have similar minor allele frequencies [5].

Linkage disequilibrium analysis did not show association between the two variants in either of the cohorts (r^2 ^= 0.005; in Pune Indians and r^2 ^= 0.007 in UK whites), indicating that the two variants fall on different haplotypes in Indians as they do in other populations.

### Effect of the APOA5 variants on triglyceride concentrations and other lipid parameters in the Pune Indian and UK white subjects

The effect of each *APOA5 *polymorphism in each group of subjects is shown in table 3. The presence of the -1131C allele (TC and CC subjects combined) is associated with higher triglyceride concentrations in the Pune Indians by 0.25 standard deviations (95% CI, 0.12–0.39; p = 0.0003) and in the Plymouth EarlyBird study UK whites by 0.5 standard deviations (95% CI, 0.11–0.89; p = 0.01) relative to TT subjects. The difference in size effect between the two groups of subjects is not significant.

**Table 3:** Association of serum triglyceride concentrations and other lipid parameters (mg/dl) with *APOA5 *genotype for -1131T>C and S19W.

		-1131T>C					S19W			
		
		TT	CT	CC	Effect on lipid (SD)*	P Value	SS	SW+WW	Effect on lipid (SD)	P Value
Triglycerides	Pune Indians	91.5 (3.1)	110.1 (4.3)	106.0 (13.1)	0.25 (0.12, 0.39)	0.0003	97.2 (2.5)	114.0 (11.0)	0.25 (-0.11, 0.60)	0.17
	UK whites	95.3 (3.8)	125.3 (12.6)	-	0.50 (0.11, 0.89)	0.01	94.4 (3.7)	123.0 (12.1)	0.41 (0.10, 0.73)	0.008
HDL	Pune Indians	41.4 (0.7)	41.2 (0.9)	37.2 (2.9)	-0.03 (-0.21, 0.15)	0.73	41.1 (0.6)	41.7 (2.4)	0.07 (-0.32, 0.47)	0.71
	UK whites	55.8 (0.95)	54.2 (2.3)	-	-0.03 (-0.49, 0.44)	0.91	56.1 (0.96)	51.9 (2.5)	-0.33 (-0.72, 0.07)	0.10
LDL	Pune Indians	96.8 (1.8)	101.6 (2.5)	100.1 (7.6)	0.11 (-0.08, 0.29)	0.25	98.2 (1.5)	103.5 (6.3)	0.16 (-0.24, 0.56)	0.44
	UK whites	115.5 (2.4)	123.9 (5.5)	-	0.26 (-0.21, 0.73)	0.27	115.5 (2.3)	121.1 (8.3)	0.05 (-0.35, 0.46)	0.79
Total Cholesterol	Pune Indians	156.6 (2.0)	164.6 (2.7)	158.6 (8.5)	0.19 (0.01, 0.36)	0.04	158.8 (1.6)	167.9 (7.1)	0.25 (-0.15, 0.64)	0.22
	UK whites	189.8 (2.5)	202.6 (5.4)	-	0.36 (-0.11, 0.83)	0.13	190.0 (2.5)	197.1 (8.6)	0.13 (-0.27–0.53)	0.52

Values are given as mean (SE), units (mg/dl); P values are based on linear regression analysis. SD = standard deviations. *Effect on triglycerides in Pune Indians is for CC+CT individuals versus TT individuals and for both studies are based on logged values. There are no -1131CC individuals in the UK white population.

The presence of the 19W allele was associated with raised triglyceride concentrations in the Pune Indians by 0.25 standard deviations (95% CI, -0.11–0.60; p = 0.17), although this did not reach significance, and in the Plymouth EarlyBird study UK whites by 0.41 standard deviations (95% CI, 0.10–0.73; p = 0.008). The 15–19% increase in the fasting triglyceride concentration associated with the inheritance of less common alleles at either of the two polymorphisms in the Pune Indians is a very similar effect size to that seen in previous studies of Caucasian subjects. This suggests that the main difference between Pune Indians and UK whites is the allele frequency rather than how much they alter the triglyceride concentration. We also performed all analyses whilst correcting for age and sex but the p values and effect sizes changed very little because genotypes were not associated with age or sex. There were no associations between the two *APOA5 *variants and either HDL, LDL or total cholesterol in either the Pune Indian or UK white subjects, although this may be due to lack of power if the effects on these lipid parameters is less than the effects on triglycerides.

We next assessed the extent of linkage disequilibrium between the *APOA5 *-1131T>C SNP and the *APOC3 SstI *SNP in Pune Indians and observed D' and r^2 ^values between these two SNPs of 0.41 and 0.07 respectively. This suggests that the highly significant association between the *APOA5 *-1131T>C variant and triglyceride concentrations is not driven by LD with the *APOC3 SstI *SNP. Further work is needed to assess more comprehensively the role of other common variations in the *APOC3 *gene in Indian subjects.

### Comparison of associations between genotype and phenotype variables on triglyceride concentrations in the Pune Indians and the UK white subjects

Regression analysis to determine the effect of different variables on triglycerides concentrations is shown in table 4. Except for the S19W variant, all the variables including age, sex, BMI, fasting glucose and insulin and -1131T>C were significantly associated with triglyceride concentrations in the Pune Indian individuals. Sex and BMI were most strongly correlated with triglyceride concentrations in the Pune Indians (r^2 ^= 0.13 and r^2 ^= 0.12 respectively, p < 0.001) whilst sex, BMI and fasting insulin were all strongly correlated with triglyceride concentrations in the UK whites (r^2 ^= 0.12, r^2 ^= 0.14 and r^2 ^= 0.16 respectively, p < 0.001). In both cohorts, the *APOA5 *alleles are associated with variation in triglyceride concentrations.

**Table 4:** Separate effects of different variables on triglyceride levels for the Asian Indians and UK whites.

Variable	Pune Indians		UK whites	
		
	Adjusted R^2^	P value	Adjusted R^2^	P value
Age	0.08	<0.001	-0.004	0.84
Sex	0.13	<0.001	0.12	<0.001
BMI	0.12	<0.001	0.14	<0.001
Fasting Glucose	0.014	0.003	0.00001	0.32
Fasting Insulin	0.06	<0.001	0.16	<0.001
HDL-Cholesterol	0.07	<0.001	0.17	<0.001
LDL-Cholesterol	0.08	<0.001	0.13	<0.001
Total Cholesterol	0.23	<0.001	0.21	<0.001
-1131T>C	0.023	0.0003	0.009	0.015
S19W	0.002	0.17	0.025	0.008
All variables together	0.26	<0.001	0.31	<0.001

Asian Indians (adults from the Pune Children Study) and UK whites (adults from the Plymouth EarlyBird Study).

## Conclusion

We conclude that the -1131C allele in *APOA5 *is considerably more common in Indo Europeans from Maharashtra (Pune Indians) than in UK white subjects and has a similar impact on triglyceride concentration. Conditions associated with hypertriglyceridaemia, such as pancreatitis, insulin resistance, type 2 diabetes and coronary heart disease are highly prevalent in Asian Indian populations. The relatively high -1131C allele frequency within Pune Indian subjects will mean it is possible to assess whether a life-long increase in triglyceride concentrations as a result of inheriting this variant increases the risk of these disorders [20].

## Abbreviations

*APOA5 *– Apolipoprotein A5

*APOA1 *– Apolipoprotein AI

*APOA4 *– Apolipoprotein AIV

*APOC3 *– Apolipoprotein CIII

SNPs – Single Nucleotide Polymorphisms

HDL – High Density Lipoprotein

LDL – Low Density Lipoprotein

OGTT – Oral Glucose Tolerance Test

LD – Linkage Disequilibrium

CV – Coefficient of Variation

HWE – Hardy Weinberg Equilibrium

CI – Confidence Intervals

BMI – Body Mass Index

## Competing interests

The author(s) declare that they have no competing interests.

## Authors' contributions

GRC, KJW, CSY, ATH, TMF all designed the genetic study; most of the practical work was carried out by KJW, GRC and PMK. The Pune Children's study was designed by CSY, ANP, AB, CHDF and CVJ. The Plymouth EarlyBird study was designed by TJW, CVJ, BSM & MNW and TMF carried out the analysis of the data. GRC, KJW, TMF and ATH wrote the first draft of the paper with all authors contributing to and approving the final version.

## Pre-publication history

The pre-publication history for this paper can be accessed here:


